# First record of *Australatyaobscura* Han & Klotz, 2015 (Decapoda, Atyidae) from the Ryukyu Islands, Japan

**DOI:** 10.3897/BDJ.7.e30507

**Published:** 2019-03-01

**Authors:** Naoto Inui, Tomoaki Maruyama, Ken Okamoto

**Affiliations:** 1 Department of Biological Sciences, Faculty of Science, The University of Tokyo, Kawasaki, Japan Department of Biological Sciences, Faculty of Science, The University of Tokyo Kawasaki Japan; 2 Department of Ecosystem Studies, Graduate School of Agricultural and Life Sciences, The University of Tokyo, Yokohama, Japan Department of Ecosystem Studies, Graduate School of Agricultural and Life Sciences, The University of Tokyo Yokohama Japan; 3 Department of Ecosystem Studies, Graduate School of Agricultural and Life Sciences, The University of Tokyo, Matsudo, Japan Department of Ecosystem Studies, Graduate School of Agricultural and Life Sciences, The University of Tokyo Matsudo Japan

**Keywords:** *
Australatya obscura
*, Atyidae, biogeography, freshwater shrimp, Ryukyu Islands

## Abstract

**Background:**

The freshwater shrimp, *Australatyaobscura* Han & Klotz, has been known only from Taiwan and Panay Island, Philippines.

**New information:**

An adult *A.obscura* was collected from a river on Ishigaki Island, Ryukyu Islands, Japan. This is the first record of the species in Japan and the northernmost specimen-supported record to date. The species is suggested as having been transported northwards by the Kuroshio Current.

## Introduction

The Ryukyu Islands are located southwest of the Japanese mainland and are the northernmost region with a tropical rainforest climate. The region is known for its high diversity of atyid shrimps which currently includes 22 species belonging to seven genera, namely *Antecaridina*, *Atyoida*, *Atyopsis*, *Caridina*, *Halocaridinides*, *Neocaridina* and *Paratya* (Cai and Shokita 2006, Chace 1983, Toyota and Seki 2014, Weese et al. 2012). In addition, an undescribed species, considered to belong to a new genus, was reported as Atyidae gen. sp. by Fujita et al. (2017). However, shrimps of the genus *Australatya* Chace, 1983 have not been previously recorded.

*Australatyaobscura* Han & Klotz, 2015 is a rare freshwater filter-feeding atyid shrimp. Although the small and numerous eggs indicate a possibly wide distribution range in South-East Asia, the species has been reported only from Taiwan and Panay Island, Philippines (Han and Klotz 2015). This study reports the first Japanese record of *A.obscura*, based on an adult specimen.

## Materials and methods

The shrimp was caught by hand net from under a stone in a fast-flowing stream of the Fukido River, Ishigaki Island, Ryukyu Islands, Japan on 11 March 2018. Its distinctive colouration clearly separated it from other filter-feeding shrimps (*Atyopsisspinipes*) collected at the time. After being photographed, the specimen was preserved in 70% ethanol and later examined under a microscope and measured using a digital caliper. The carapace length was measured from the postorbital margin to the posterior margin of the carapace. The specimen was deposited in the Kanagawa Prefectural Museum of Natural History (registration number: KPM-NH 3213).

## Taxon treatments

### Australatya obscura

Han and Klotz, 2015

#### Materials

- **Type status:**Other material. **Occurrence:** catalogNumber: KPM-NH 3213; recordedBy: Naoto Inui; individualCount: 1; sex: female; lifeStage: adult; reproductiveCondition: non-ovigerous; disposition: in collection; **Taxon:** scientificName: Australatyaobscura; order: Decapoda; family: Atyidae; genus: Australatya; **Location:** higherGeography: East Asia; islandGroup: Ryukyu Islands; island: Ishigaki island; country: Japan; countryCode: Japan/JP; stateProvince: Okinawa; county: Ishigaki; municipality: Nosoko; locality: the Fukido river; verbatimCoordinates: 24°28.92’N, 124°14.17’E; **Identification:** identifiedBy: Tomoaki Maruyama and Naoto Inui; dateIdentified: 2018-03-11; **Event:** samplingProtocol: hand net; year: 2018; month: 3; day: 11; habitat: freshwater river; **Record Level:** institutionCode: Kanagawa Prefectural Museum of Natural History (KPM); basisOfRecord: Preserved Specimen

#### Diagnosis

**Figure 1.**
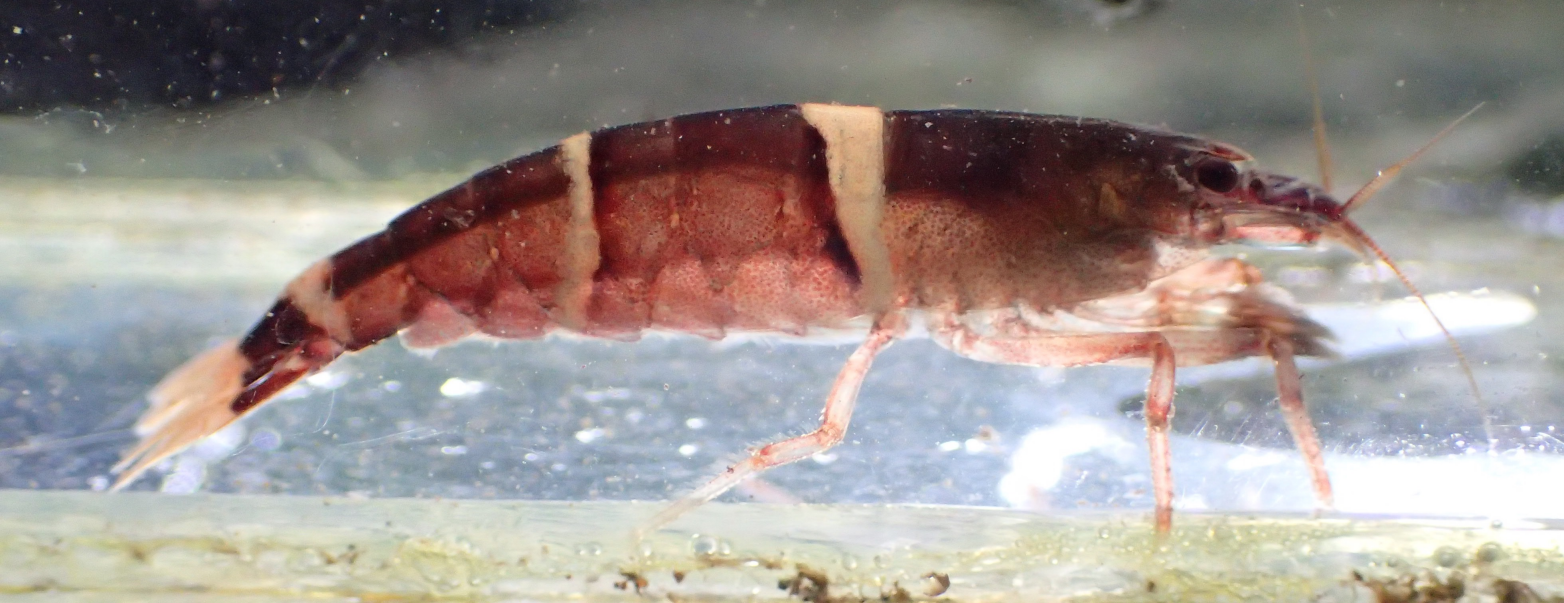
Photograph of the live specimen of *Australatyaobscura* (registration number: KPM-NH 3213).

The specimen was a non-ovigerous adult female with carapace length 6.5 mm and rostral formula 0/4. The third maxilliped was slender, lacking terminal spiniform seta. The merus of the third pereiopod had a row of plumose setae. The live specimen had black and white vertical bands on the abdominal segments (Fig. 1). The morphological features and live colouration of the specimen agreed well with the original description of *Australatyaobscura* (Han and Klotz 2015).

#### Distribution

Japan: Ishigaki Island (this study); Taiwan; Philippines: Panay Island

#### Habitat

**Figure 2.**
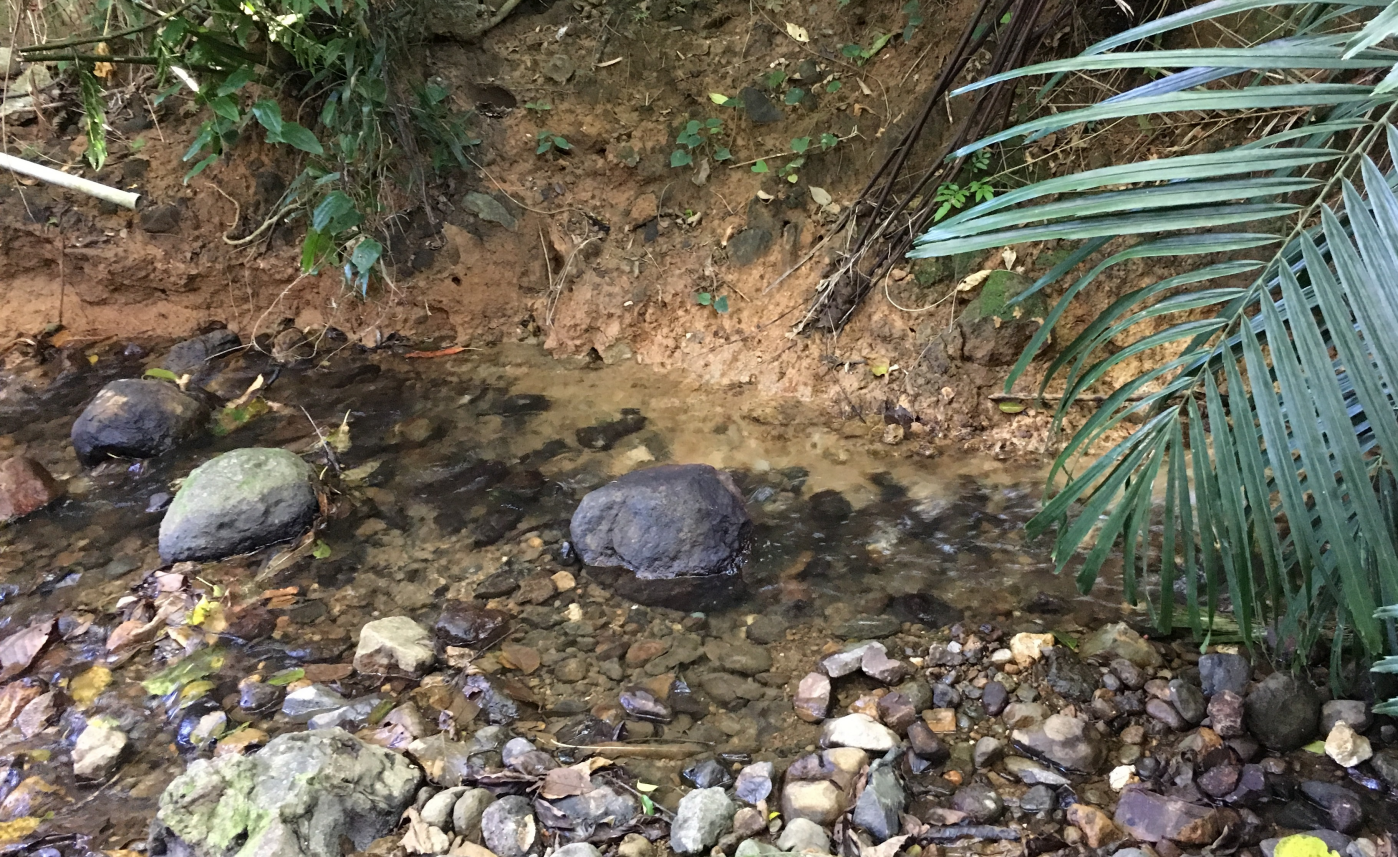
The collection site of *Australatyaobscura* in the Fukido River, Ishigaki Island, Ryukyu Islands.

The collection site was a wooded section of the middle reaches of the Fukido River (Fig. 2). Sediment at the site comprised pebbles and cobbles. The water temperature was 14.8°C and depth about 0.2 m. Other atyid shrimps and palaemonid prawns caught in the same habitat included *Atyopsisspinipes* (Newport, 1847), *Caridinalaoagensis* Blanco, 1939, *C.macrodentata* Cai and Shokita, 2006, *C.typus* H. Milne Edwards, 1837, *Macrobrachiumlar* (Fabricius, 1798), *M.latimanus* (von Martens, 1868), *M.placidulum* (De Man, 1892), *Macrobrachium* sp. 1 (Japanese name: “Chura-tenaga-ebi”) and *Macrobrachium* sp. 2 (Japanese name: “Kasuri-tenaga-ebi”).

## Discussion

**Figure 3.**
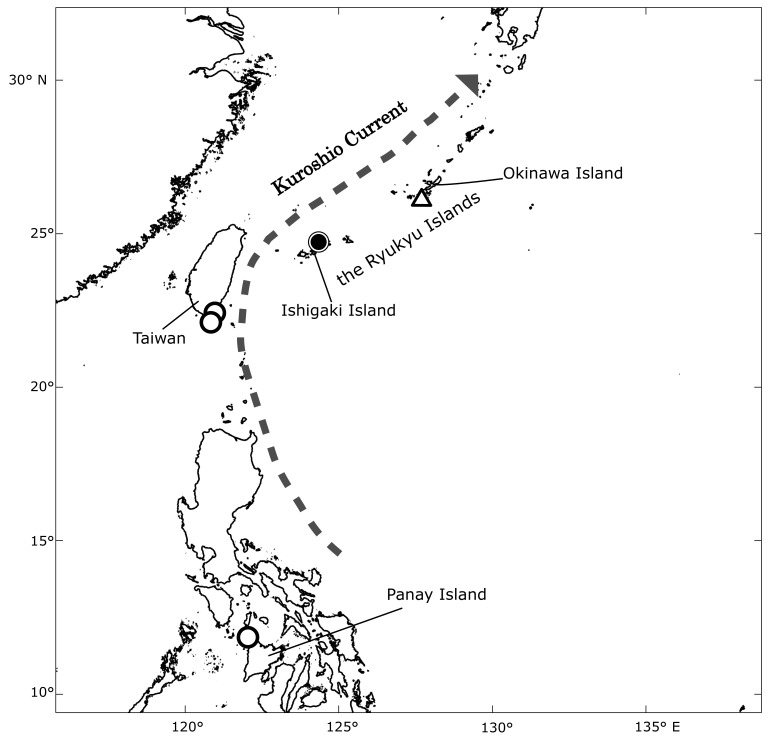
Map of distribution records of *Australatyaobscura*. Solid circle indicates present specimen-based record on Ishigaki island; open circles, literature records (Han and Klotz 2015); open triangle, photographic record from Okinawa island. Map drawn using Mirone (Luis 2007).

The present report is the first record of the species in Japan and the northernmost record based on a voucher specimen. However, a photograph of an immature *A.obscura* collected on Okinawa Island, Ryukyu Islands was taken by Tsuhako (2016). Known localities of *A.obscura* are shown in Fig. 3. It is believed that individuals of the species found in the Ryukyu Islands were transported from southern habitats by the Kuroshio Current during their larval stage. Considering that the current specimen was an adult and must have overwintered, it seems likely that the species could gain a foothold in the Ryukyu islands.

The new standard Japanese name "Hime-oni-numa-ebi" is proposed for *A.obscura*. The body size of *A.obscura* is relatively small compared with other *Atya*-like species in Japan, the new Japanese name reflecting its small size.

## Supplementary Material

XML Treatment for Australatya obscura
